# PI3K/AKT/mTOR and sonic hedgehog pathways cooperate together to inhibit human pancreatic cancer stem cell characteristics and tumor growth

**DOI:** 10.18632/oncotarget.5055

**Published:** 2015-10-05

**Authors:** Narinder Sharma, Rajesh Nanta, Jay Sharma, Sumedha Gunewardena, Karan P. Singh, Sharmila Shankar, Rakesh K. Srivastava

**Affiliations:** ^1^ Department of Pharmacology, Toxicology and Therapeutics, and Medicine, University of Kansas Medical Center, Kansas City, KS, 66160, USA; ^2^ Celprogen Inc. Torrance, CA 90503, USA; ^3^ Department of Molecular and Integrative Physiology, University of Kansas Medical Center, Kansas City, KS, 66160, USA; ^4^ Division of Preventive Medicine, School of Medicine, University of Alabama at Birmingham, Birmingham, Al, 35205, USA; ^5^ Kansas City VA Medical Center, Kansas City, MO, 66128, USA; ^6^ Department of Pathology, University of Missouri-School of Medicine, Kansas City, MO, 64108, USA

**Keywords:** pancreatic cancer, PI3K/AKT/mTOR, sonic hedgehog, cancer stem cell, Gli

## Abstract

Cancer stem cells (CSCs) play major roles in cancer initiation, progression, and metastasis. It is evident from growing reports that PI3K/Akt/mTOR and Sonic Hedgehog (Shh) signaling pathways are aberrantly reactivated in pancreatic CSCs. Here, we examined the efficacy of combining NVP-LDE-225 (PI3K/mTOR inhibitor) and NVP-BEZ-235 (Smoothened inhibitor) on pancreatic CSCs characteristics, microRNA regulatory network, and tumor growth. NVP-LDE-225 co-operated with NVP-BEZ-235 in inhibiting pancreatic CSC's characteristics and tumor growth in mice by acting at the level of Gli. Combination of NVP-LDE-225 and NVP-BEZ-235 inhibited self-renewal capacity of CSCs by suppressing the expression of pluripotency maintaining factors Nanog, Oct-4, Sox-2 and c-Myc, and transcription of Gli. NVP-LDE-225 co-operated with NVP-BEZ-235 to inhibit Lin28/Let7a/Kras axis in pancreatic CSCs. Furthermore, a superior interaction of these drugs was observed on spheroid formation by pancreatic CSCs isolated from *Pan^kras/p53^* mice. The combination of these drugs also showed superior effects on the expression of proteins involved in cell proliferation, survival and apoptosis. In addition, NVP-LDE-225 co-operated with NVP-BEZ-235 in inhibiting EMT through modulation of cadherin, vimentin and transcription factors Snail, Slug and Zeb1. In conclusion, these data suggest that the combined inhibition of PI3K/Akt/mTOR and Shh pathways may be beneficial for the treatment of pancreatic cancer.

## INTRODUCTION

Pancreatic cancer has very poor prognosis due to its late diagnosis, silent nature, low surgical resectability rates, and resistance to standard chemotherapy and radiation [1]. Pancreatic cancer is a lethal malignancy with a 5-year survival rate less than 6% and is ranked as the fourth leading cause of cancer-related deaths in the United States [2]. Currently, there are very limited treatment options for pancreatic cancer, therefore there is an urgent need to develop novel and more efficient drugs. Currently, there are very limited therapeutic options for pancreatic cancer, therefore new drugs and novel treatment approaches are urgently needed.

Despite the initial effectiveness of conventional therapies against cancer, metastases recurrence and emergence are major causes of therapeutic failure in cancer patients. These therapies mainly target the differentiated and proliferative cells that comprise the bulk of the tumor. The relapse of cancer is attributed to a small population of cancer stem cells (CSC) residing within the tumor. In addition to resistance to typical therapies, CSCs are reported to have inherently high tumor-initiating potential, which is implicated in tumor relapse, driving primary tumor growth as well as establishment of metastases by CSCs [3, 4].

Hedgehog (Hh) is an intercellular signaling molecule (polypeptide) that was discovered in drosophila [5]. Hh signaling pathway plays a crucial role in embryonic development from invertebrates to mammals [6, 7]. Sonic hedgehog (Shh) is one of the members of the Hh family [8], and studies have demonstrated that abnormal activation of the Shh pathway due to genomic alterations leads to an increase in cell survival and metastasis in cancer cells [9, 10]. In fact, higher Hh levels along with enhanced expression of Hh pathway target genes has been identified in different cancers, such as pancreatic cancer, gastric cancer, upper gastrointestinal cancer, and prostate cancer [10]. Shh ligands bind to its receptor Patched (Patch) and causes internalization Patch, which in its active form inhibits Smoothened (Smo). Smo positively regulates the activation of Glioma-associated (Gli) family of zinc finger transcription factors, which ultimately translocate to nucleus to induce gene transcription [11, 12]. Expression levels of Gli have been interrelated with the expression levels of the Shh pathway all together, suggesting Gli transcriptional activity is a direct indicator of Shh pathway activity [13]. Oncogenic activating mutations in the components of the Hh signaling pathway have been reported in several cancer [14–16]. In contrast, inactivating mutations have been found in Patch1 associated with Gorlin-Goltz Syndrome. Gorlin-Goltz Syndrome patients develop basal cell carcinomas and have higher risk of developing rhabdomyosarcoma and medulloblastoma [17]. In agreement with the role of Hh signaling in tumor development, a variety of drugs have been developed in the recent years to block this signaling pathway in cancer cells including Hh antagonist SANTs1–4, Gli inhibitor GANT-61, and smoothened inhibitor NVP-LDE-225 [18–20].

The Phosphoinositide 3-kinase-AKT-mammalian target of rapamycin (PI3K-AKT-mTOR) pathway plays an important role in various cellular processes, including growth regulation, and apoptosis [21]. This pathway is a commonly hyper activated in cancer and promotes cell survival, proliferation and cancer progression [22]. mTOR is an important target of Akt and exist in two distinct functional complexes, mTOR complex I and mTOR complex II [23]. mTOR-Complex I regulates the expression of ribosomal protein S6 kinase (S6K) and eukaryotic translation initiation factor 4E-binding-protein-1 (4E-BP1),which in turn regulate the expression of Bad, cyclin D1, and Bcl-2 [23, 24]. Inhibition of mTOR alone results in cancer relapse due to up-regulation of PI3K activity. Therefore dual inhibitors of PI3K and mTOR may be beneficial for a long-term treatment to inhibit cell growth. NVP-BEZ-235 is a dual inhibitor of PI3K as well as mTOR complex I and complex II and it has shown anti-proliferative effects in various cancers [25, 26]. NVP-BEZ-235 has been shown to radiosensitize prostate cancer cells under normoxic and hypoxic conditions [27]. It is currently being tested in phase I/II clinical trials in cancer patients [28].

miRNAs are small noncoding RNAs and have been shown to regulate gene expression [29, 30]. It has been shown that let-7 family of miRNAs plays an important role in cell differentiation, development and tumor suppression [31]. Lin28 is a developmentally regulated RNA binding protein which regulates the biogenesis of let-7 micro-RNA family [32, 33]. Lin28 acts as an oncogene and promotes carcinogenesis [34–37]. The Lin28/Let-7 pathway affects many cellular processes including the regulation of CSCs, differentiation and glycolytic metabolism [34, 38–41]. Furthermore, Lin28/let7 axis may be exploited to overcome the radioresistance of human cancers having activated K-Ras signaling [42]. Overexpression of LIN28 correlates with poor outcome [43, 44], therefore drugs that impact the Lin28/Let-7 pathway could be beneficial in treating cancer patients.

The CSCs demonstrate the epithelial-to-mesenchymal transition (EMT) program that has well-established roles in promoting an invasive and metastatic cancer [45, 46]. In the process of EMT, cancerous cells lose contact with surrounding cells, undergo major changes in their cytoskeleton, and acquire a mesenchymal-like structure which assist them with increased invasive and migratory abilities [47, 48]. The process of EMT occurs through downregulation of the epithelial adhesion protein E-cadherin (CDH1), which is repressed by the transcriptional regulators Zeb1, Zeb2, Twist1, Snail and Slug, which also regulate various other epithelial-related genes [49–52]. Many signaling pathways regulate the process of EMT including the Shh pathway [53].

The purpose of this study was to examine the interactive effects of NVP-LDE-225 and NVP-BEZ-235 on characteristics of pancreatic CSCs in culture and inhibition of tumor growth in NOD/SCID IL2Rγ null mice. Data obtained from this study demonstrate that the combination of NVP-LDE-225 and NVP-BEZ-235 was superior than single agent alone in inhibiting the self-renewal of pancreatic CSCs by suppressing the expression of pluripotency-maintaining factors (Nanog, Oct-4, Sox-2 and c-Myc) and transcription of Gli. These drugs also co-operated in inhibiting EMT by suppressing transcription factors Snail, Slug and Zeb1. The combination of these drugs was also superior in inhibiting the pancreatic CSC tumor growth in NOD/SCID IL2Rγ null mice than single agent alone. These data clearly suggest that the combination of NVP-LDE-225 and NVP-BEZ-235 is a potential therapeutic strategy for the treatment of pancreatic cancer by targeting CSCs.

## RESULTS

### NVP-BEZ-235 inhibits the PI3/mTOR/Akt pathway, and NVP-LDE-225 inhibits the components of the Shh pathway in pancreatic CSCs

**Figure 1:**
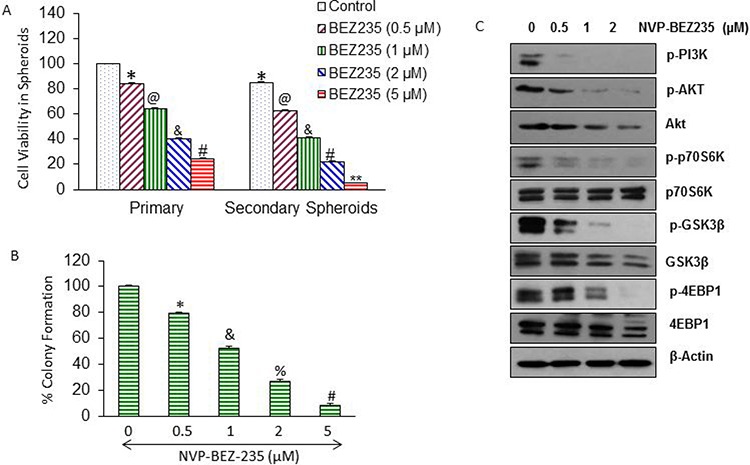

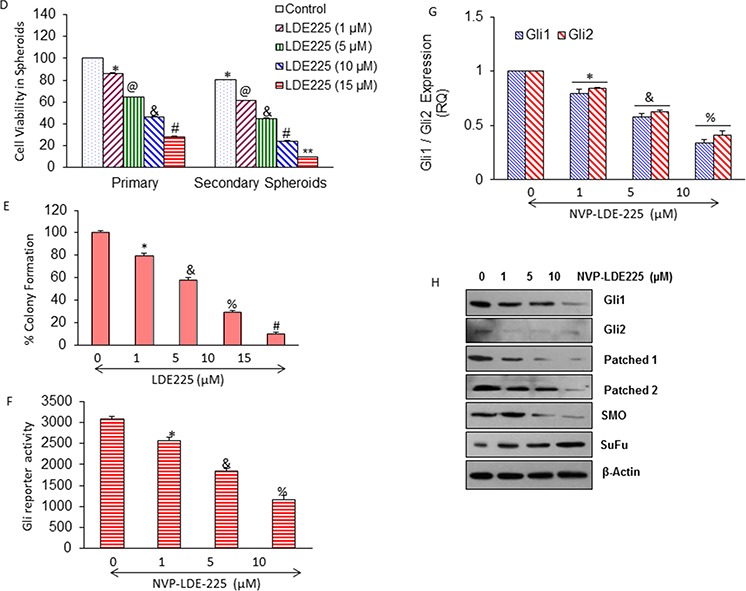
Regulation of PI3/Akt/mTOR and Shh pathways by NVP-BEZ-235, and NVP-LDE-225 in pancreatic CSCs, respectively **A.** CD44^+^CD24^+^ESA^+^ CSCs were isolated from human primary pancreatic tumors, and treated with NVP-BEZ-235 (0–5 μM) for 7 days. At the end of incubation period, spheroids were collected, reseeded and treated with NVP-BEZ-235 for another week to obtain secondary spheroids. *, @, &, #, and ** = significantly different from control, *P* < 0.05. **B.** Pancreatic CSCs were seeded in soft agar and treated with NVP-BEZ-235 (0–5 μM) for 21 days. At the end of incubation period, numbers of colonies were counted. *, &, %, and # = significantly different from control, *P* < 0.05. **C.** Pancreatic CSCs were treated with different concentrations of NVP-BEZ-235 (0–2 μM) and expression of phospho-Akt, Phospho-PI3K, Phospho-p70S6K, Phospho-GSK3Kβ, Phospho-4EBP1, total Akt, p70S6K, total GSK3Kβ, and total 4EBP1 was analyzed by Western blot analysis. β-Actin was used as a loading control. **D.** Pancreatic CSCs were treated with NVP-LDE-225 (0–15 μM) for 7 days. At the end of incubation period, spheroids were collected, reseeded and treated with NVP-LDE-225 for another week to obtain secondary spheroids. *, @, &, #, and ** = significantly different from control, *P* < 0.05. **E.** Pancreatic CSCs were seeded in soft agar and treated with NVP-LDE-225 (0–15 μM) for 21 days. At the end of incubation period, numbers of colonies were counted. *, &, %, and # = significantly different from control, *P* < 0.05. **F.** CSCs were transduced with Gli-responsive GFP/firefly luciferase viral particles (pGreen Fire1-Gli with EF1, System Biosciences), and treated with NVP-LDE-225 (0–10 μM) for 36 h. Gli reporter activity was measured by luciferase assay. **G.** Pancreatic CSCs were treated with NVP-LDE-225 (0–10 μM) for 48 h. The expression of Gli1 and Gli2 was measured by qRT-PCR. Data represent mean ± SD. * &, and % = significantly different from control, *P* < 0.05. **H.** Effect of NVP-LDE-225 on components of Shh pathway. Pancreatic CSCs were treated with different concentrations of NVP-LDE-225 (0–10 μM) for 48 h, and expressions of Gli1, Gli2, Patched1, Patched2, smoothened and SuFu were determined by Western blot analysis. β-Actin was used as a loading control.

PI3/mTOR/Akt pathway plays a significant role in pancreatic carcinogenesis [62, 63]. We therefore examined the effects of NVP-BEZ-235 on cell viability in spheroids formed by CD44^+^CD24^+^ESA^+^ CSCs (Fig. 1A). Primary spheroids were grown in suspension for one week, whereas for secondary spheroids, CSCs from primary spheroids were reseeded, treated and grown for another week. NVP-BEZ-235 inhibited cell viability in primary and secondary spheroids in a dose-dependent manner. Similarly, NVP-BEZ-235 inhibited colony formation of pancreatic CSCs in a dose-dependent manner (Fig. 1B).

We first examined the effect of NVP-BEZ-235 on the components of PI3K/Akt/mTOR pathway in pancreatic CSCs (Fig. 1C). Pancreatic CSCs treated with different concentrations of NVP-BEZ-235 showed decrease in the levels of phospho-PI3K, phospho-Akt, phospho-p70S6K, phospho-GSK3Kβ, and phospho-4EBP1; whereas the expression of total p70S6K was not altered in drug treated samples as compare to untreated control of pancreatic CSCs. Surprisingly, we have seen a slight decline in the expression of total Akt, total GSK3Kβ, and total 4EBP1 levels at higher doses of treatment with NVP-BEZ-235, which may be due to degradation of proteins during apoptosis.

Shh pathway is constitutively active in pancreatic cancer and plays a significant role in CSC's survival [64]. We therefore examined the effects of NVP-LDE-225 on cell viability in spheroids formed by pancreatic CSCs (Fig. 1D). NVP-LDE-225 inhibited cell viability in primary and secondary spheroids in a dose-dependent manner. Similarly, NVP-LDE-225 inhibited colony formation of pancreatic CSCs in a dose-dependent manner (Fig. 1E).

We next examined the effects of NVP-LDE-225 on Gli reporter activity by luciferase assay, and Gl1 and Gl2 expression by q-RT-PCR (Fig. 1F and 1G). NVP-LDE-225 inhibited Gli reporter activity. Gli is a transcriptional target of its own, because Gli1 and 2 contain Gli binding sites. NVP-LDE-225 inhibited the expression of Gli1 and Gli2 as measured by q-RT-PCR. We next examined the effect of NVP-LDE-225 on the components of Shh pathway by measuring the expression of Gli1, Gli2, patched 1, patched 2, Smo and Sufu by Western blot analysis. Pancreatic CSCs were treated with NVP-LDE-225 (0–2 μM) for 48 hrs. As shown in Fig. 1H, the expression of Gli1, Gli2, pathched1, pathched2 and smoothened (SMO) was decreased and the expression of SuFu was increased by treating with different concentrations of NVP-LDE-225. These protein profiles clearly show that NVP-BEZ-235 inhibits PI3/mTOR/Akt pathway, and NVP-LDE-225 inhibits Shh pathway in pancreatic CSCs.

### NVP-LDE-225 cooperates with NVP-BEZ-235 in inhibiting cell viability and colony and spheroid formation, and in inducing apoptosis in pancreatic CSCs

**Figure 2:**
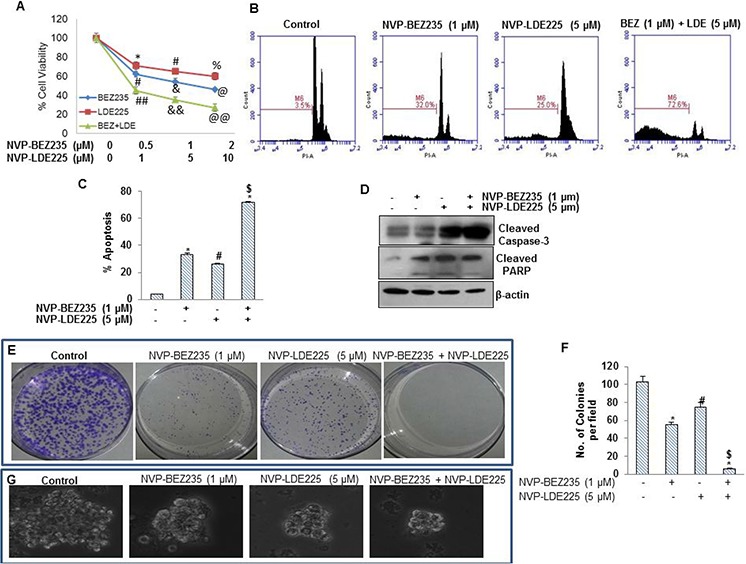

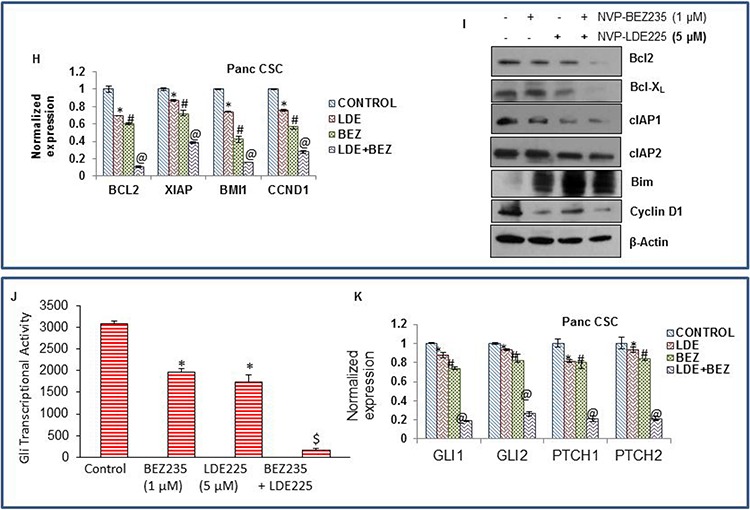
Effect of NVP-LDE-225, NVP-BEZ-235 and their combination on cell viability, apoptosis, and colony and spheroid formation, and protein expression **A.** Effects LDE-225, BEZ-235 and their combination on cell viability. Pancreatic CSCs were seeded in 96 well plates and treated with NVP-LDE-225 (5 μM), NVP-BEZ -235 (1 μM) and their combination for 48 h. Thereafter, cell viability was measured by XTT assay. Viable cells were quantified. **B.** Pancreatic CSCs were treated with NVP-LDE-225 (5 μM), NVP-BEZ -235 (1 μM) and their combination for 48 h. Thereafter, cell apoptosis was measured by PI staining. Data are representative of 3 independent experiments. **C.** Quantification of apoptotic cells. Data represent mean (*n* = 4) ± S.D. *, #, and $ = significantly different from control, *P* < 0.05. **D.** Expression of cleaved caspase-3 and cleaved PARP in pancreatic CSCs treated with NVP-LDE-225 (5 μM), NVP-BEZ -235 (1 μM) and their combination. Cells were treated with these drug(s) for 48 h, and the expression of cleaved caspase-3 and PARP was analyzed by Western blot analysis. **E.** Pancreatic CSCS were treated with NVP-LDE-225, NVP-BEZ-235 and their combination for 48 h, thereafter, fresh media added to cells. After 10 days, colonies were observed by staining the plates with 0.5% crystal violet strain. **F.** Quantification of pancreatic CSCs colonies. Data represent mean (*n* = 4) ± S.D. *, #, and $ = significantly different from control, *P* < 0.05. **G.** Effects of NVP-LDE-225, and/or NVP-BEZ -235 on spheroid formation by pancreatic CSC. Pancreatic CSCs were seeded in suspension and treated with NVP-LDE-225 (5 μM), NVP-BEZ -235 (1 μM) and their combination for 7 days. Spheroid images were obtained by light microscopy. **H.** qRT-PCR analysis of expression profile for Bcl-2, XIAP, BMI and CCND1 in pancreatic CSCs after treatment with NVP-LDE-225 (5 μM), NVP-BEZ -235 (1 μM) and their combination for 36 h. HK-GAPD was used as the endogenous normalization control. Data represent mean (*n* = 4) ± S.D. *, #, and @ = significantly different from control, *P* < 0.05. **I.** Pancreatic CSCs were treated with NVP-LDE-225, NVP-BEZ-235 and their combination for 48 h, and the expression of Bcl2, Bcl-X_L_, cIAP1, cIAP2, Bim, cyclin D1, and β-actin (loading control) was measured by Western blot analysis. **J.** Pancreatic CSCs were transduced with Gli-responsive GFP/firefly luciferase viral particles (pGreen Fire1-Gli with EF1, System Biosciences). After transduction, culture medium was replaced and CSCs were treated with BEZ235 (1 μM) and/or LDE225 (5 μM) for 24 h. Gli reporter activity was measured as we described [82]. **K.** Pancreatic CSCs were treated with NVP-LDE-225 (5 μM), NVP-BEZ-235 (1 μM) or their combination for 36 h. RNA was extracted and expressions of Gli1, Gli2, Patched1 (PTCH1), and Patched2 (PTCH2) were measured by qRT-PCR. Data represent mean (*n* = 4) ± S.D. *, #, and @ = significantly different from control, *P* < 0.05.

We first examined the interactive effects of NVP-LDE-225 and NVP-BEZ-235 on viability of pancreatic CSCs by XTT assay. As shown in Fig. 2A, NVP-LDE-225 and NVP-BEZ-235 alone inhibited cell viability in a dose-dependent manner. The NVP-LDE-225 cooperated with NVP-BEZ-235 in inhibiting viability of pancreatic CSCs. The combination of NVP-LDE-225 and NVP-BEZ-235 was superior than single agent alone.

In order to check the synergetic effect of NVP-LDE-225 and NVP-BEZ-235 on the induction of apoptosis in pancreatic CSCs, we treated the cells with these drugs alone and with their combination for 48 h, and analyzed apoptosis by PI staining assay (Sub G0 cells) using flow cytometry. NVP-LDE-225 and NVP-BEZ-235 alone induced apoptosis in pancreatic CSCs (Fig. 2B). Furthermore, NVP-LDE-225 cooperated with NVP-BEZ-235 in inducing apoptosis in pancreatic CSCs. Quantitative analysis of apoptotic cells clearly demonstrates that the combination of NVP-LDE-225 and NVP-BEZ-235 was superior than single agent alone in inducing apoptosis in pancreatic CSCs (Fig. 2C). Further, the combination of NVP-LDE-225 and NVP-BEZ-235 was superior than single agent alone in inducing cleavage of caspase3 into its active form (Fig. 2D). Poly-ADP ribose polymerase (PARP) is a substrate of caspase 3 therefore; we next analyzed the cleavage of PARP by Western blot analysis. These drugs showed co-operative effects on cleavage of PARP as well. We next examined the interactive effects of NVP-LDE225 and NVP-BEZ235 on colony formation of pancreatic CSCs. NVP-LDE225 and NVP-BEZ235 alone inhibited colony formation of pancreatic CSCs (Fig. 2E and 2F), but when used in combination these drugs inhibited colony formation to a higher extent. These data demonstrate the synergetic anti-proliferative and apoptotic effects of NVP-LDE225 and NVP-BEZ235 on pancreatic CSCs.

Spheroid formation in suspension is one of the characteristics of CSCs. We therefore examined the interactive effects of NVP-LDE225 and NVP-BEZ235 on spheroid formation of pancreatic CSCs. NVP-LDE225 and NVP-BEZ235 alone inhibited spheroid formation of pancreatic CSCs (Fig. 2G). Furthermore, the combination of NVP-LDE225 and NVP-BEZ235 was more effective in inhibiting spheroid formation than single agent alone. These data suggest that NVP-LDE225 and NVP-BEZ235 cooperates together to inhibit cell viability, and colony and spheroid formation, and to induce apoptosis.

### Regulation of cell survival-, cell cycle- and apoptosis-related genes by NVP-LDE-225 and/or NVP-BEZ-235 in pancreatic cancer stem cells

Based upon results obtained from apoptosis and cell viability, we next examined the expression of key regulatory proteins behind this process. We examined the effects of NVP-LDE-225 and/or NVP-BEZ-235 on the expression of Bcl-2, XIAP, BMI1 and CCND1 by qRT-PCR (Fig. 2H). NVP-LDE-225 and NVP-BEZ-235 alone inhibited the expression of Bcl-2, XIAP, BMI1 and CCND1 in pancreatic CSCs. Furthermore, NVP-LDE-225 cooperated with NVP-BEZ-235 in inhibiting the expression of these genes.

We further explored the combination effects of these drugs on the expression of proteins that regulate cell survival, cell proliferation, apoptosis and cell cycle by the Western blot analysis (Fig. 2I). NVP-LDE-225 and NVP-BEZ-235 alone inhibited the expression of Bcl-2, Bcl-X_L_, cIAP1, cIAP2, and cyclin D1 in pancreatic CSCs. NVP-LDE-225 and/or NVP-BEZ-235 upregulated the expression of Bim in pancreatic CSCs. Furthermore, the combination of NVP-LDE-225 and NVP-BEZ-235 was superior than single agent alone in inhibiting the expression of Bcl-2, Bcl-X_L_, cIAP1, cIAP2 and cyclin D1. These data suggest that inhibition of PI3K/Akt/mTOR and Shh pathways together may be clinically beneficial for modulation of genes which regulate cell proliferation, survival, apoptosis and cell cycle in pancreatic CSCs.

### The combination of NVP-LDE-225 and NVP-BEZ-235 was superior than single agent alone in suppressing Shh pathway

Aberrant activation of PI3K/mTOR and Shh signaling pathways play important roles in tumorigenesis and progression of tumors [65–67]. The expression of Gli can be regulated by both canonical (Shh) and non-canonical (PI3K/Akt) pathways. Inhibition of PI3K/AKT pathway can inhibit Gli transcription activity through non-canonical pathway [68]. We first examined the interactive effects of BEZ235 and LDE225 on Gli reporter activity in pancreatic CSCs (Fig. 2J). The combination of BEZ235 and LDE225 was superior than single agent alone in inhibiting Gli reporter activity. The expressions of Gli, Patched 1 and Patched 2 are transcriptionally regulated by Gli. We therefore examined the effects of NVP-LDE-225 and/or NVP-BEZ-235 on the expression of Gli1, Gli2, Patched1 and Patched2 in pancreatic CSCs. NVP-LDE-225 and NVP-BEZ-235 alone inhibited the expression of Gli1, Gli2, Patched1 and Patched2 as measured by qRT-PCR (Fig. 2K). Furthermore, the combination of NVP-LDE-225 and NVP-BEZ-235 had superior inhibitory effects on the expression of Gli1, Gli2, Patched1 and Patched2. These data suggest that BEZ235 and LDE225 can inhibit self-renewal capacity of pancreatic CSCs by targeting Shh pathway.

### NVP-LDE-225, NVP-BEZ-235 and their combination inhibit cell viability in spheroids formed by pancreatic CSCs isolated from humans, and *Kras^G12D^*; Trp53*^LSL-R172H/+^*PDAC (Pan*^kras/p53^)* mice

**Figure 3:**
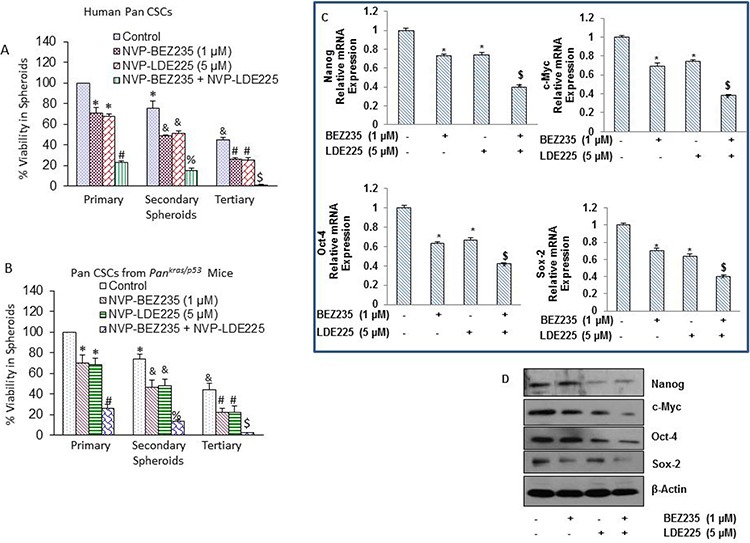

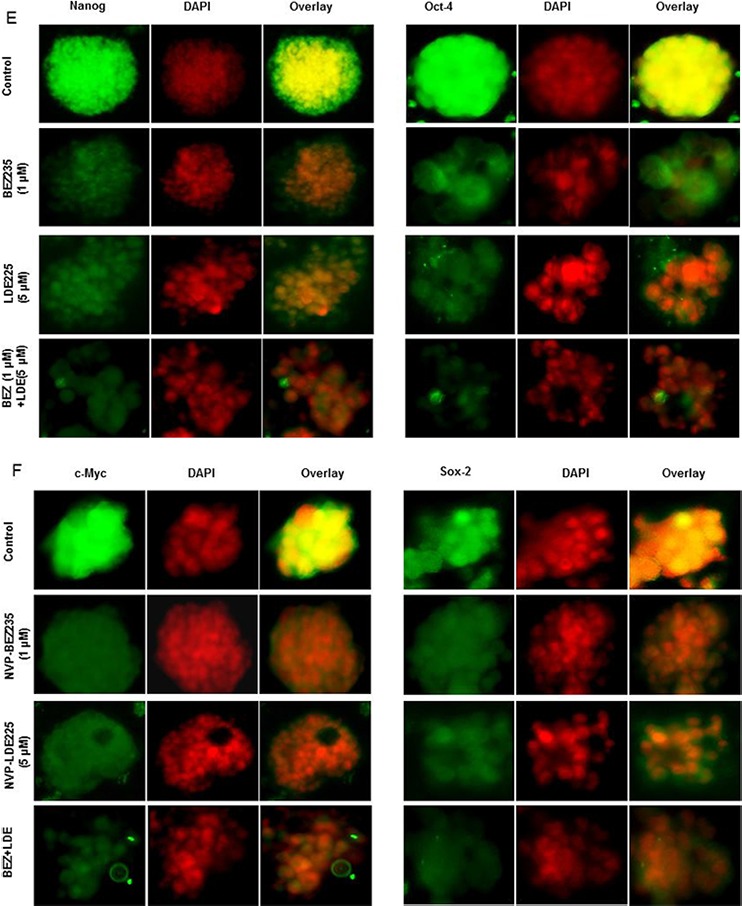
NVP-LDE-225, NVP-BEZ-235 and their combination inhibit spheroid formation by CSCs isolated from pancreas of human and Kras^G12D^; Trp53^LSL-R172H/+^ PDAC (Pan^kras/p53^) mice, and differentially regulates genes involved in self-renewal and pluripotency of pancreatic CSCs **A.** Human pancreatic CSCs isolated from primary tumors were treated with NVP-LDE-225, NVP-BEZ-235 and their combination for 7 days to obtain primary spheroids. At the end of incubation period, spheroids were collected, reseeded and treated with NVP-LDE-225 and/or NVP-BEZ-235 for another week to obtain secondary spheroids. Secondary spheroids were collected, reseeded and treated with NVP-LDE-225 and/or NVP-BEZ-235 for another week to obtain tertiary spheroids. Cell viability in spheroids was measured by trypan blue assay at the end of 7, 14 and 21 days. Data represent mean ± SD. *, &, #, % and $ = significantly different from control, *P* < 0.05. **B.** Pancreatic CSCs isolated from Pan^kras/p53^ mice were treated with NVP-LDE-225, NVP-BEZ-235 and their combination for 7 days to obtain primary spheroids. At the end of incubation period, spheroids were collected, reseeded and treated with NVP-LDE-225 and/or NVP-BEZ-235 for another week to obtain secondary spheroids. Secondary spheroids were collected, reseeded and treated with NVP-LDE-225 and/or NVP-BEZ-235 for another week to obtain tertiary spheroids. Cell viability in spheroids was measured by trypan blue assay at the end of 7, 14 and 21 days. Data represent mean ± SD. *, &, #, % and $ = significantly different from control, *P* < 0.05. **C.** Pancreatic CSCs were treated with NVP-LDE-225, NVP-BEZ-235 and their combination for 36 h, and expressions of Nanog, Oct-4, c-Myc and Sox-2 were quantified by qRT-PCR. HK-GAPDH was used as the endogenous normalization control. Data represent mean (*n* = 4) ± SD. * = significant difference from control, *P* < 0.05. $ = significant difference from control or single agent alone, *P* < 0.05. **D.** Protein expression of Nanog, c-Myc, Oct-4 and Sox-2. Pancreatic CSCs were treated with NVP-LDE-225, NVP-BEZ-235 and their combination for 48 h, and the expression of Nanog, c-Myc, Oct-4 and Sox-2 was determined by the Western blot analysis. β-actin was used as a loading control. **E.** and **F.** Immunohistochemical examination of Nanog, Oct-4, c-Myc, and Sox2 in pancreatic CSCs treated with NVP-LDE-225, NVP-BEZ-235 and their combination. Pancreatic CSCs were grown in suspension and treated with above mentioned drugs for 48 h. Spheroids formed by pancreatic CSCs were fixed and IHC was performed as described in Material and Methods.

Spheroid formation in suspension is one of the characteristics of CSCs [69]. Kras^G12D/p53^ mice mimic pancreatic cancer development in humans [60]. We have recently reported that pancreatic CSCs isolated from Kras^G12D^ mice are phenotypically similar and also respond to anticancer drugs as pancreatic CSCs isolated from humans [54, 57, 58, 69–72]. Since CSCs play a major role in cancer initiation, progression, metastasis and drug resistance, they can be used to assess the response of anticancer drugs. We next examined the effects of NVP-LDE-225, NVP-BEZ-235 and their combination on growth of human pancreatic CSCs by measuring cell viability in spheroids (Fig. 3A). NVP-LDE-225 and NVP-BEZ-235 alone inhibited cell viability of primary, secondary and tertiary spheroids formed by human pancreatic CSCs. Furthermore, NVP-LDE-225 cooperated with NVP-BEZ-235 in inhibiting cell viability of primary, secondary and tertiary spheroids. These data suggest that the combination of NVP-LDE-225 and NVP-BEZ-235 may be beneficial for the treatment of pancreatic cancer by targeting CSCs.

We next examined whether the pancreas of *Pan^kras/p53^* mouse harbor CSCs, and whether they are capable of self-renewing and respond to BEZ235 and LDE225 *in vitro* (Fig. 3B). BEZ235 and LDE225 inhibited the self-renewal capacity of pancreatic CSCs isolated from *Pan^kras/p53^* mice in a cooperative manner, as measured by formation of primary, secondary and tertiary spheroids in suspension, and cell viability in those spheroids. These data suggest that combined inhibition of PI3K/mTOR and Shh pathways is superior than single pathway inhibition in suppressing the self-renewal capacity of pancreatic CSCs isolated from *Pan^kras/p53^* mice.

### NVP-LDE-225, and NVP-BEZ-235 cooperate together to regulate the expression of pluripotency maintaining factors in pancreatic CSCs

Sox-2, Nanog, c-Myc, and Oct-4 are the transcription factors which regulate the self-renewal capacity of CSCs. Inhibition of these genes retards cell proliferation and inhibits tumor growth. Therefore, we analyzed the expression of these transcription factors in pancreatic CSCs treated with NVP-LDE-225 and NVP-BEZ-235 alone and in combination. Pancreatic CSCs were exposed to NVP-LDE-225 and NVP-BEZ-235 alone and with their combination for 36 h and then expression of Nanog, Oct-4, c-Myc and Sox-2 was measured by qRT-PCR. NVP-LDE-225 or NVP-BEZ-235 inhibited the expression of Nanog, Oct-4, c-Myc and Sox-2 at transcriptional level in pancreatic CSCs; further, even higher inhibition was observed in the expression of these factors in samples treated with combination of these drugs (Fig. 3C).

We further validated the data obtained from qRT-PCR by the Western blot analysis, where NVP-LDE-225 co-operated with NVP-BEZ-235 in inhibiting the expression of Nanog, Oct-4, c-Myc and Sox-2 in pancreatic CSCs (Fig. 3D). In addition, we studied the interactive effects of these drugs on the expression of Nanog, Oct-4, c-Myc and Sox-2 in pancreatic CSC spheroids by immunocytochemistry (Fig. 3E and 3F), where these drugs inhibited the expression of Nanog, Oct-4, c-Myc and Sox-2 in pancreatic CSC spheroids. These data suggest that inhibition of the Shh pathway and PI3/Akt/mTOR pathways can suppress the self-renewal capacity of pancreatic CSCs by inhibiting the factors required for maintaining pluripotency of pancreatic CSCs.

### NVP-LDE-225, NVP-BEZ-235 and their combination inhibit epithelial-mesenchymal transition of pancreatic CSCs

**Figure 4:**
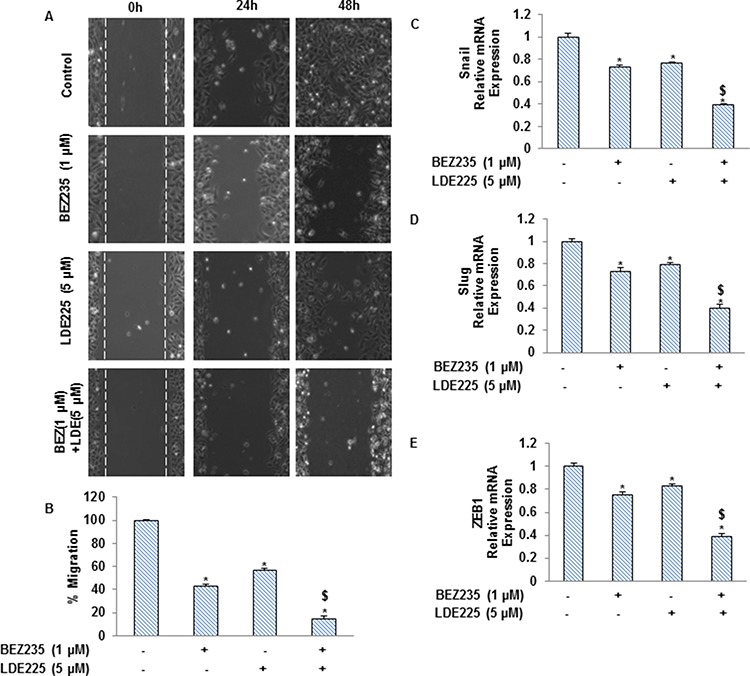
NVP-LDE-225, NVP-BEZ-235 and their combination regulate the pancreatic CSC motility and migration, and the expression of EMT-related genes **A.** Motility Assay. *In vitro* motility of pancreatic CSCs was observed by using scratch technique. CSCs were grown in monolayer, scratched and treated with NVP-LDE-225, NVP-BEZ-235 and their combination for 48 h. Data are representative of three independent experiments. **B.** Transwell migration assay. Pancreatic CSCs were plated in the top chamber of the transwell and treated with NVP-LDE-225, NVP-BEZ-235 and their combination for 24 h. cells migrated to the lower chambered were fixed with methanol, stained with crystal violet and counted. Data represent mean (*n* = 4) ± S.D. * = significantly different from control, *P* < 0.05. $ = significantly different from control or single drug treatment, *P* < 0.05. **C–E.** Pancreatic CSCs were treated with NVP-LDE-225, NVP-BEZ-235 and their combination for 36 h. After incubation, the expression of snail, slug and Zeb1 was measured by qRT–PCR. Data represent mean (*n* = 4) ± S.D. * = significantly different from control, *P* < 0.05. $ = significantly different from control or single drug treatment, *P* < 0.05.

As CSCs appear to have a significant role in early metastasis, we pursued to examine the effects of NVP-LDE-225, NVP-BEZ-235 and their combination on EMT of pancreatic CSCs (Fig. 4). These drugs inhibited cell motility and migration of pancreatic CSCs, and their combination showed superior effects in suppressing CSC's motility and migration (Fig. 4A and 4B). We also analyzed the expression of transcription factors involved in the process of EMT (Fig. 4C–4E). NVP-LDE-225 and NVP-BEZ-235 alone inhibited the expression of Snail, Slug and Zeb1 as analyzed by qRT-PCR. Furthermore, the combined inhibitory effects of NVP-LDE-225 and NVP-BEZ-235 on Snail, Slug and Zeb1 expression was significantly higher than single agent alone. These findings suggest that combination of NVP-LDE-25 and NVP-BEZ-235 can inhibit events behind early metastasis with pancreatic CSCs.

### Regulation of microRNA network by NVP-BEZ-235 and/or NVP-LDE-225 in human pancreatic cancer stem cells

**Figure 5:**
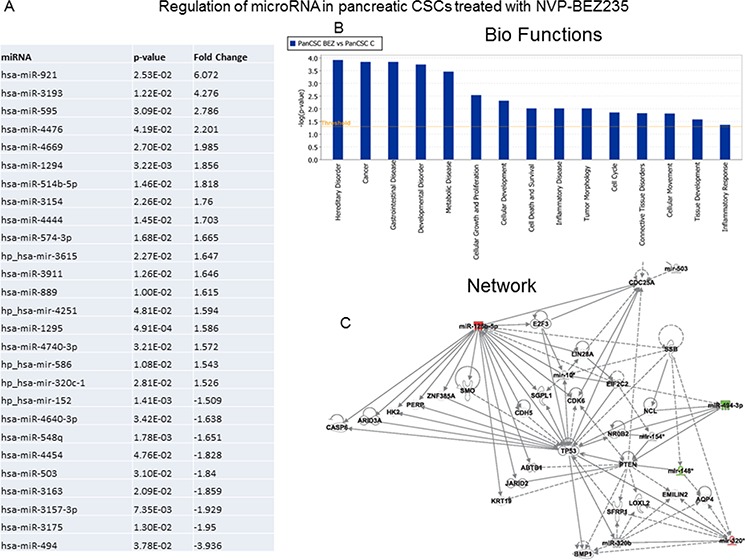

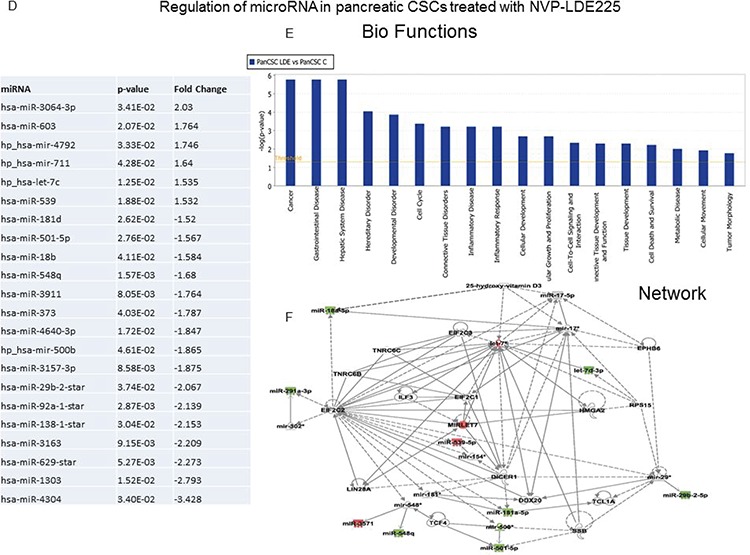

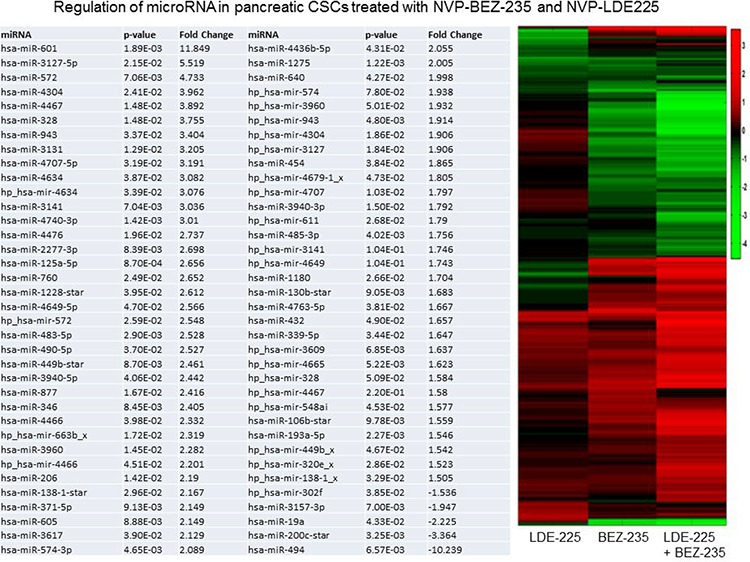

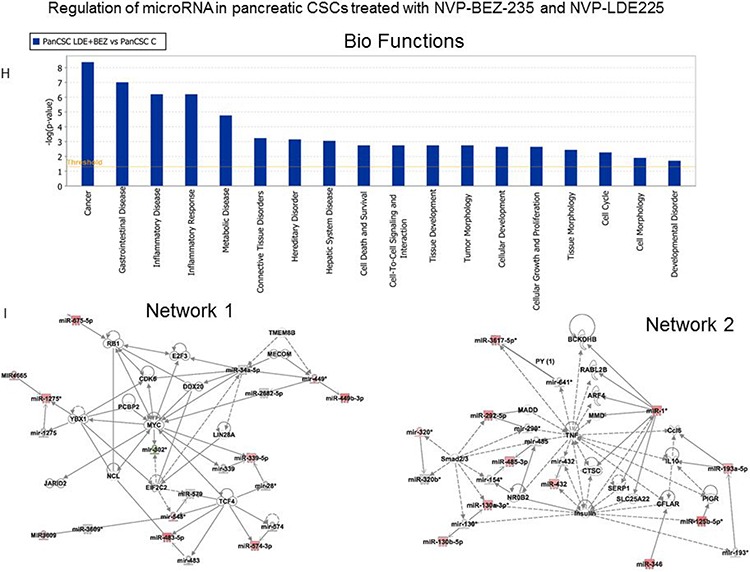
Regulation of miRNAs in human pancreatic CSCs treated with NVP-BEZ-235 and/or NVP-LDE-225 **A.** Pancreatic CSCs were treated with NVP-BEZ-235 (1 μM) for 36 h. RNA was isolated and miRNA array (Affimetrix gene Chip) analysis was performed. Expression of selected miRNAs. *P*-values and fold change are also shown. **B.** Bio-function analysis. **C.** Netwrok analysis. **D.** Pancreatic CSCs were treated with NVP-LDE-225 (5 μM) for 36 h. RNA was isolated and miRNA array (Affimetrix gene Chip) analysis was performed. Expression of selected miRNAs. P-values and fold change are also shown. **E.** Bio-function analysis. **F.** Netwrok analysis. **G.** Pancreatic CSCs were treated with NVP-BEZ-235 (1 μM) and NVP-LDE-225 (5 μM) for 36 h. RNA was isolated and miRNA array (Affimetrix gene Chip) analysis was performed. Left panel, Expression of selected miRNAs. P-values and fold change are also shown. Right panel, Heat Map of miRNA arrays from NVP-LDE-225- and/or NVP-BEZ-235- treated pancreatic CSCs. **H.** Bio-function analysis. **I.** Netwrok analysis.

Recent studies have demonstrated that microRNAs (miRNAs) play an important role in regulation of stem cell self-renewal, proliferation, and differentiation, and their aberrances cause the formation of CSCs which promote cancer initiation, progression and metastasis. We therefore examined the effects of NVP-BEZ-235 and/or NVP-LDE-225 on the expression of miRNA in pancreatic CSCs, and also performed bio-functional analysis and identified regulatory network based on global expression of miRNAs. NVP-BEZ-235 (NVP-BEZ-235 vs control) induced the expression of 18 miRNAs and inhibited the expression of 9 miRNAs in pancreatic CSCs (Fig. 5A). Based on bio-function analysis, NVP-BEZ-235 regulated those miRNAs which control hereditary disorder, cancer gastrointestinal disorder, developmental disorder, metabolic disorder, cellular growth and proliferation, cellular development, cell death and survival, inflammatory disease, tumor morphology, cell cycle, connective tissue disorders, cellular movement, tissue development, and inflammatory response (Fig. 5B). Network analysis has identified the regulation of mainly TP53, CASP6, ARID3A, HK2, PERP, ZNF385A, SMO, E2F3, CDC25A, LIN28A, EIF2C2, CDK6, PTEN, BMP1, LOXL2, SFRP1, EMILN2, KRT19, JARID2, and ABTB4 (Fig. 5C).

NVP-LDE-225 (NVP-LDE-225 vs control) induced the expression of 5 miRNAs and inhibited the expression of 16 miRNAs in Pancreatic CSCs (Fig. 5D). Based on bio-function analysis, NVP-LDE-225 regulated those miRNAs which control cancer, gastrointestinal disease, hepatic system disease, hereditary disorder, developmental disorder, cell cycle, connective tissue disorders, inflammatory disease, inflammatory response, cellular development, cellular growth and proliferation, cell-to-cell signaling and interaction, connective tissue development and function, tissue development, cell death and survival, metabolic disease, cellular movement and tumor morphology (Fig. 5E). Network analysis has identified the regulation of mainly EIF2C2, EIF2C1, EIF2C8, TNRC6B, TNRC6C, LIN28A, ILF3, TCF4, DICER1, 25-hydroxy vitamin D3, TCL1A and SSB (Fig. 5F).

The combination of NVP-BEZ-235 and NVP-LDE-225 (NVP-BEZ-235 and NVP-LDE-225 vs control) induced the expression of 50 miRNAs and inhibited the expression of 4 miRNAs in Pancreatic CSCs (Fig. 5G). Heat map demonstrated the differential expression of miRNA expression in NVP-LDE-225 and/or NVP-BEZ-235-treated pancreatic CSCs. Based on bio-function analysis, NVP-BEZ-235 and NVP-LDE-225 together modulated those miRNAs which regulate cancer, gastrointestinal disease, inflammatory disease, inflammatory response, metabolic disease, connective tissue disorders, hereditary disorder, hepatic system disease, cell death and survival, cell-to-cell signaling and interaction, tissue development, tumor morphology, cellular development, cellular growth and proliferation, tissue morphology, cell cycle, cell morphology and developmental disorders (Fig. 5H). We have identified two main regulatory network in cells treated with the combination of NVP-BEZ-235 and NVP-LDE-225 (Fig. 5I). Analysis of network 1 has identified the regulation of YBX1, PCB2, JARID2, MYC, RB1, CDK6, E2F3, LIN28A, TCF4, EIF2C2, NCL, MECOM and TMEM8B. Furthermore analysis of network 2 has identified Smad2/3, MADD, TNF, insulin, CFLAR, RABL2B, SERP1, IL-10, CTSC, SERP1, and SLC225A22. Taking together, our findings define that NVP-BEZ-235 and NVP-LDE-225 might play a critical role in inhibiting the stem cell survival, self-renewal, and metastasis through regulation of miRNAs.

### BEZ235 and LDE225 regulate Lin28/Let7a/Kras axis in pancreatic CSCs

**Figure 6:**
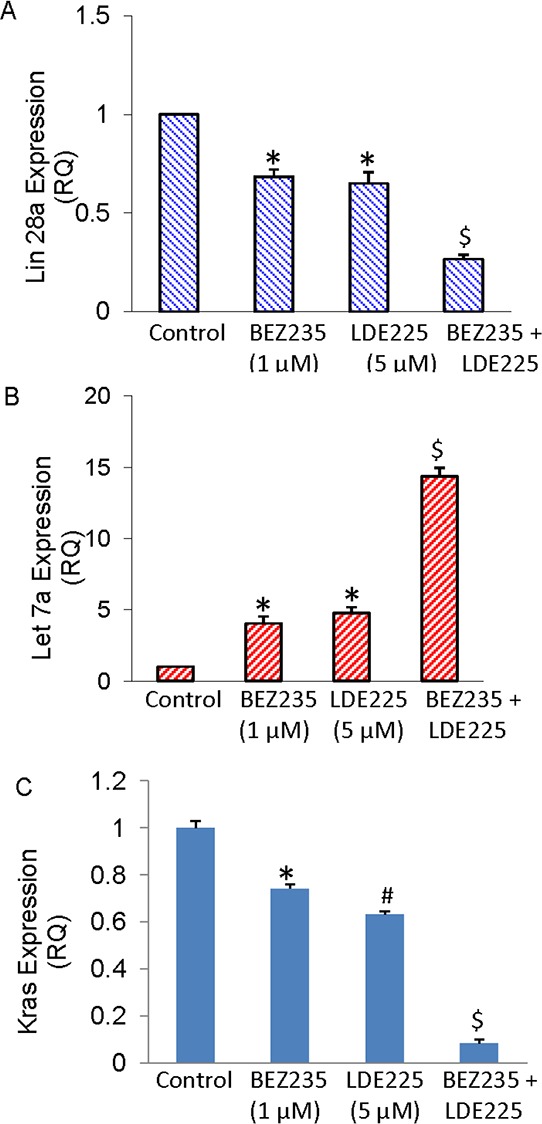
BEZ235 and LDE225 regulate Lin28/Let7a/Kras axis in pancreatic CSCs **A–C.** Expression of Lin28a, Let7a and Kras. Pancreatic CSCs were treated with BEZ235 (1 μM) and/or LDE225 (5 μM) for 36 h. RNA was extracted and the expression of Lin28a, Let7a and Kras was measured by qRT-PCR. Data represent mean (*n* = 4) ± S.D. * and $ = significantly different from control, *P* < 0.05.

Lin28/Let-7 pathway regulates several biological processes, such as differentiation of stem cell, invasion and metastasis, aerobic glycolysis, and tumorigenesis [34, 38–40, 42–44]. We therefore examined whether treatment of CSCs with NVP-BEZ-235 and/or NVP-LDE-225 regulate the expression of Lin28, let7 and Kras. NVP-BEZ-235 and NVP-LDE-225 alone inhibited the expression of Lin28a and Kras, and induced the expression of Let7a (Fig. 6A–6C). The combination of NVP-BEZ-235 and NVP-LDE-225 was more effective in inhibiting the expression of Lin28a and Kras, and inducing the expression of Let7a. These data suggest that targeting of Lin28/Let7/Kras axis by NVP-BEZ-235 and NVP-LDE-225 may be a useful strategy for the treatment of pancreatic cancer.

### The combination of NVP-LDE-225 and NVP-BEZ-235 was superior than single agent alone in inhibiting pancreatic CSC tumor growth in NOD/SCID IL2Rγ null mice

**Figure 7:**
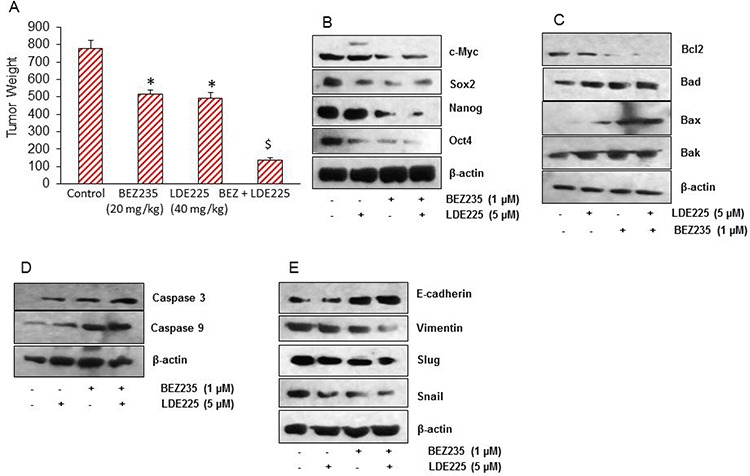
NVP-LDE-225, NVP-BEZ-235 and their combination inhibit pancreatic CSC tumor growth in NOD/SCID IL2Rγ null mice **A.** Effects of NVP-LDE-225 and/or NVP-BEZ-235 on pancreatic CSC tumor growth in NOD/SCID IL2R γ null mice. Tumor-bearing nude mice were injected with vehicle or NVP-LDE-225 (40 mg/kg), NVP-BEZ-235 (20 mg/kg) and their combination intraperitoneally, five times per week for 4 weeks. Tumor weights were measured at the end of the experiments. Data represent mean ± S.D. * = significantly different from control, *P* < 0.05. $ = significantly different from control or single drug treatment, *P* < 0.05. **B.** Expression of pluripotency maintaining factors. Tissue lysates were prepared from tumor tissues isolated from control and NVP-LDE-225, NVP-BEZ-235 and their combination treated mice. Western blot analysis was performed to measure the expression of c-Myc, Sox-2, Nanog, and Oct-4. **C.** Expression of Bcl-2 family members. Tissue lysates were prepared and the expression of Bcl2, Bad, Bax, and Bak was measured by the Western blot analysis. β-actin was used as a loading control. **D.** Expression of Caspase 3 and Caspase 9. Tissue lysates were prepared and the expression of Caspase 3 and Caspase 9 was measured by the Western blot analysis. β-actin was used as a loading control. **E.** Expression of E-cadherin, Vimentin, Slug, and Snail. Tissue lysates were prepared and the expression of E-cadherin, Vimentin, Slug, and Snail was measured by the Western blot analysis. β-actin was used as a loading control.

Since NVP-LDE-225 and/or NVP-BEZ-235 inhibited cell viability, spheroid and colony formation, and induced apoptosis in pancreatic CSCs, we next sought to examine their effects on pancreatic CSC xenografted tumor growth in NOD/SCID IL2Rγ null mouse model. Pancreatic CSCs were injected subcutaneously into NOD/SCID IL2Rγ null mice. After tumor formation, mice were treated with saline, NVP-LDE-225 (40 mg/kg), NVP-BEZ-235 (20 mg/kg) or combination of both drugs intraperitoneally 5 days/week for 4 weeks. NVP-LDE-225, and NVP-BEZ-235 alone inhibited tumor growth (as judged by tumor weight), and their combination had superior effects on tumor growth inhibition (Fig. 7A). Mice weight were also checked during the course of the study, which remained normal and therefore these drugs alone or in combination had no adverse effect on the health of the mice (data not shown).

Since NVP-LDE-225 and/or NVP-BEZ-235 inhibited pancreatic CSC's tumor growth in mice, we next sought to examine the molecular mechanisms of action of these drugs. We first measured the expression of proteins involved in maintaining pluripotency of pancreatic CSCs by the Western blot analysis. As shown in Fig. 7B, NVP-LDE-225 and NVP-BEZ-235 alone inhibited the expression of c-Myc, Sox-2, Nanog and Oct-4 in tumor samples. However, the combination of these drugs was more effective in inhibiting the expression of these proteins than single agent alone.

Further, we also analyzed the expression of Bcl-2 protein family in tumor samples by Western blot analysis (Fig. 7C). The expression of Bcl-2 was reduced whereas the expression of Bad, Bax, and Bak was found to be higher in tumor samples treated with these drugs. The amount of cleaved caspase 3, and cleaved caspase9 were also found to increase in tumor samples treated with NVP-LDE-225 and/or NVP-BEZ-235 (Fig. 7D). These data suggest that NVP-LDE-225 and/or NVP-BEZ-235 inhibited CSC's tumor growth by modulating Bcl-2 family members and activating caspases.

The process of metastasis initiation requires invasion, which is enabled by EMT. Carcinoma cells in primary tumor lose cell-cell adhesion mediated by E-cadherin repression and break through the basement membrane, and enter in bloodstream through intravasation. Therefore, we examined the expression of EMT-related proteins by measuring the expression of E-cadherin, Vimentin, Slug and Snail. The expression of E-cadherin was higher in tumor samples derived from NVP-LDE-225 or NVP-BEZ-235 treated mice. Furthermore, the E-cadherin expression was even higher in tumor samples derived from mice treated with the combination of NVP-LDE-225 and NVP-BEZ-235 (Fig. 7E). Conversely, the expression of Vimentin was found to be lower in tumor samples derived from NVP-LDE-225 or NVP-BEZ-235 treated mice, and even lesser in tumors where combination of these drugs was used. Since NVP-LDE-225 and NVP-BEZ-235 were effective in regulating the expression of E-cadherin and Vimentin. We also measured the expression of EMT-related transcription factors Snail, and Slug. As analyzed by the Western blot analysis, the expression of both of these transcription factors was decreased in the tumor tissue derived from NVP-LDE-225 and/or NVP-BEZ-235 treated mice. These data from mice tumor tissue indicate that both NVP-LDE-225 and NVP-BEZ-235 can lower the potential of early metastasis events, and combination of both drugs can lower this process to even higher extant.

## DISCUSSION

Tumors arise from a small population of cancer cells (usually less than 5% of cells of a tumor) that have properties of adult stem cells, particularly the ability to self-renew and multilineage differentiation. These properties bring CSCs at the apex of tumor biology architecture. The CSCs are quite resistant to standard chemotherapy and ionizing radiation therapy, and play a major role in relapse of cancer. Therefore, there is an urgent need to develop new chemotherapeutic drugs or develop unique combination of existing drugs to achieve the inhibition on characteristics of CSCs. In the present study, we have demonstrated for the first time that combination of NVP-LDE-225 and NVP-BEZ-235 inhibits pancreatic CSCs characteristics and xenografted tumor growth in NOD/SCID mice. Combination of these drugs has synergetic effect in the induction of apoptosis through caspase-3 activation, and cleavage of PARP. Inhibition of Shh and PI3K/AKT pathways together may be a novel strategy for the treatment of pancreatic cancer.

The PI3K/Akt pathway contributes to regulating a variety of extracellular signals and plays an important role in tumor survival and cell proliferation and inhibition of apoptosis [21]. Akt is shown to be active in human pancreatic cancer tissues and Akt inhibitors such as ZSTK474 inhibited pancreatic cancer cell growth [73, 74]. NVP-BEZ-235 has been shown to inhibit both PI3K/Akt and mTOR signaling and inhibits tumor growth in various cancers [25, 26] and is undergoing in phase I/II clinical trials for use in solid tumors [28]. The PI3K/Akt/mTOR pathway is involved in providing stimulation for cell proliferation, inhibition of apoptosis and it is has been assessed that this pathway is dysregulated in at least 50% of all cancer types. Our data from this study clearly show that NVP-BEZ-235 specifically inhibits the components of PI3K/Akt/mTOR pathway by reducing the expression of phospho-Akt, phospho-mTOR, and phospho-p70S6 kinase.

The Shh pathway has many roles in developmental processes; regulates tissue homeostasis, and maintains stem cell populations. But in addition to its role in normal developmental processes, the Shh pathway dysregulation has been shown to cause and sustain cancer growth [75]. Excessive amount of Shh ligand has been detected in many tumors of the upper gastrointestinal tract [76] and Shh has been shown to be a critical mediator in the initiation of pancreatic cancer, its progression, and metastasis [65]. Recently, we have revealed that various components of Shh pathway are highly expressed in human pancreatic cancer cell lines and pancreatic CSCs and anticancer agents inhibited pancreatic CSC characteristics and xenografted tumor growth in mice [57, 71]. In this study, we verified that pancreatic CSCs consistently express various components of the Shh signaling pathway, including Gli1, Gli2, Patched-1 and Patched-2, suggesting that the Shh pathway is one of the ‘core’ signaling pathways in pancreatic CSCs.

In the present study, our data shows that NVP-LDE-225/NVP-BEZ-235 alone or in combination inhibited pro-survival proteins, Bcl-2 and Bcl-X_L_, and pro-apoptotic proteins, Bak and Bax, in pancreatic CSCs. Bcl-2 family proteins, which have either pro- or anti-apoptotic activities, and regulate apoptosis, tumorigenesis and cellular responses to anti-cancer therapy. XIAPs inhibit apoptosis that is induced by overproduction of caspases, and XIAP binds to caspase 3, 7 and 9 and inhibits their activity [77]. Combination of NVP-LDE-225 and NVP-BEZ-235 inhibited the expression of XIAP, survivin, cIAP1 and cIAP2.

Pan^ras/p53^ mice have concomitant endogenous expression of transgenic mutant Kras^G12D^ and p53^R172^ in the pancreas. These mice (about 100%) develop pancreatic ductal adenocarcinomas with metastasis in the liver, lung, and adrenals [61]. Pan^kras/p53^ mice mimic pancreatic cancer development in humans with similar genetic and morphological alterations. NVP-LDE-225 and NVP-BEZ-235, alone or in combination, inhibited spheroid formation by pancreatic CSCs isolated from Pan^kras/p53^ mice, suggesting the inhibition of these PI3K/Akt/mTOR and Shh pathways can be exploited in pancreatic cancer harboring mutations in Kras and p53.

Let-7 and Lin28 regulate several biological processes, such as differentiation of stem cell, invasion and metastasis, aerobic glycolysis, and tumorigenesis [34, 38–40, 42–44]. The tumor-suppressor let-7 miRNA family has been shown to be universally down-regulated in CSCs, because of abundant expression of the regulatory gene Lin28. Low let-7 levels resulted in up-regulation of oncogenes including MYCN, AURKB and Lin28 itself, the latter through a direct feedback mechanism. Let-7 is required for normal gene expression in the context of embryonic development and oncogenesis. Lin28B/Let7 regulates key CSC properties by modulating the expression of Oct-4 / Sox-2 and determines the efficiency by which normal human oral keratinocytes could be reprogrammed to iPSC [38]. The overexpression of let-7a or inhibition of Lin28 suppressed expression of K-Ras, and radiosensitized cancer cells [42]. We have demonstrated that the Lin28/Let-7/Kras axis is regulated by NVP-LDE-225 and NVP-BEZ-235 in pancreatic CSCs. Let-7 may inhibit Kras, whereas Lin28 protein could control the PI3k/AKT pathway [41]. Lin28 has been implicated in the repression of Let-7 and mediates both glucose metabolism and embryonic stem cell differentiation [41, 78]. A recent study has reported the existence of a feedback loop involving NF-κB, Lin28/Let-7 and TNF-α in transformed cells [79, 80]. These studies suggest that Lin28/Let-7/Kras pathway may be exploited to control pancreatic CSC characteristics and tumor growth.

Metastatic growth requires the dissemination of cancerous cells from the primary tumor, followed by their reestablishment in a secondary site. The EMT is critical for development of many tissues and organs in the developing embryo, and numerous embryonic events, carcinogenesis and metastasis. Signals within tumor microenvironment trigger cancer cells to acquire an invasive phenotype through EMT. Similar to cells that undergo EMT during normal development, carcinoma cells switch from a sessile, epithelial phenotype to a motile by losing cell-cell contacts, changes in the cytoskeleton, and acquire a mesenchymal-like phenotype endowing them with increased invasive and migratory abilities [47, 81]. Increasing evidence suggests that EMT plays an important role during malignant tumor progression. Various key molecules present in the tumor microenvironment serve as cytosolic bridges between receptors on the cell surface and nuclear transcription factors to induce EMT [48, 52]. Moreover, many of these molecules have also been found to be aberrantly activated and/or overexpressed in human cancers [51]. Snai1 (Snail), Snai2 (Slug), Zeb1 are EMT regulators which act by repressing the Cdh1 gene encoding E-cadherin. In the present study, NVP-BEZ-235 and NVP-LDE-225 cooperated together to inhibit EMT by pancreatic CSCs.

Our results in xenografted tumor demonstrated that NVP-BEZ-235 and NVP-LDE-225 acted in a co-operative manner to inhibit tumor growth and induce cleavage of caspase-3 and caspase-9. Cleavage of caspase-3 and caspase-9 by these drugs would indicate the induction of apoptosis in tumor tissues. NVP-LDE-225 and NVP-BEZ-235 inhibited the self-renewal capacity of pancreatic CSCs isolated from human and Pan^kras/p53^ mice *in vitro*, and CSC's tumor growth in NOD/SCID IL2Rϒ null mice, and synergetic effect was observed when both drugs were used in combination. EMT inhibition was associated with suppression of transcription factors Zeb1, Snail and Slug, and mesenchymal marker vimentin, and upregulation of epithelial marker E-cadherin in pancreatic CSCs. In summary, our findings suggest that combined inhibition of PI3K/Akt/mTOR and Shh pathways will be beneficial for the treatment of pancreatic cancer by targeting CSCs, and further study of these pathway inhibition in the clinical setting is warranted.

## EXPERIMENTAL PROCEDURES

### Reagents and cell culture

NVP-LDE225 and NVP-BEZ-235 were obtained from Chemie Tek (Indianapolis, IN). Antibodies used against Gli1, Gli2, phospho-Akt, Akt, caspase-3, PARP, E-Cadherin, N-Cadherin, Vimentin, Sox-2, Oct-4, Nanog, c-Myc, phospho-Akt, phospho-PI3K, phospho-p70S6K, phospho-GSK3Kβ, phospho-4EBP1, Akt, p70S6K, GSK3Kβ, 4EBP1, Slug, snail, Snail, patched 1, patched2, Smo, Sufu Bcl-XL, Bax, Bak, Bcl2, and β-actin were purchased from Cell Signaling Technology, Inc. (Danvers, MA). Enhanced chemiluminescence (ECL) Western blot detection reagents were purchased from Thermo Fisher Scientific Corporation (Waltham, MA).

Isolation and characterization of CD44^+^CD24^+^ESA^+^ pancreatic CSCs have been described elsewhere [54]. Pancreatic CSCs were grown in well-defined stem cell culture medium [54–56].

### Cell viability and apoptosis assays

Cell viability was determined by the XTT assay as described elsewhere [55, 57]. Briefly, pancreatic CSCs (5 × 10^4^) were incubated with 1 μM of NVP-BEZ-235, 5 μM of NVP-LDE-225, and combination of NVP-LDE-225 (5 μM) and NVP-BEZ-235 (1 μM) in 250 μl of culture medium in 96-well plate for 48 h. 50 μl of freshly prepared XTT-PMS labeling mixture was added to the cell culture. The absorbance was measured at 450 nm with correction at 650 nm. The cell viability was expressed as OD (OD450–OD650).

The apoptosis of pancreatic CSCs was determined by FACS analysis of propidium iodide (PI)-stained cells. Briefly, pancreatic CSCs were grown in 6 well plate and NVP-LDE-225, NVP-BEZ-235 and their combination were added in the media. After 48 h of incubation, cells were trypsinized, washed with PBS, resuspended in 200 μl PBS with 10 μl RNAase (10 mg/ml) and incubated at 37°C for 30 min. At the end of incubation, 50 μl PI solution was added and cells were analyzed for apoptosis using a flow cytometry (Accuri C6 flow cytometer, BD Biosciences, San Jose, CA).

### Motility assay

Cell motility assay was performed as described elsewhere [55, 57]. In brief, pancreatic CSCs were grown in monolayer and then a scratch was made with a pipette tip. After washing twice with PBS, CSCs were treated with or without NVP-LDE-225 and/or NVP-BEZ-235. Pancreatic CSCs migrate into the scratch area as single cells from the confluent sides. The width of the scratch gap was viewed under the microscope in four separate areas. Three replicate wells from a six-well plate were used for each experimental condition.

### Transwell migration assay

Cell migration assay was performed as described elsewhere [55, 57].

### Tumor spheroid assay and Immunocytochemistry

Tumor spheroid assay was performed as described elsewhere [55, 57]. In brief, pancreatic CSCs were grown in six-well ultralow attachment plates (Corning Inc.) at a density of 1000 cells/ml in well-defined pancreatic CSC medium at 37°C in a humidified atmosphere of 95% air and 5% CO_2_. NVP-LDE-225 (5 μM), NVP-BEZ-235 (1 μM) or their combination was added to each well. Similarly, for secondary spheroids, CSCs from spheroids were reseeded and treated with drugs for another 7 days. For tertiary spheroids, CSCs from spheroids were reseeded and treated with drugs for another 7 days. Immunocytochemistry of pancreatic CSCs was performed as we described elsewhere [58].

### RNA isolation and mRNA expression analysis by qRT-PCR

RNA isolation and qRT-PCR analysis were performed as described elsewhere [55, 57].

### MicroRNA array analysis

The expression levels of expressed miRNAs were measured using Affymetrix GeneChip miRNA 3.0 arrays. Only probe sets of human small-RNAs in the array were used for the analysis. They constituted 1,733 human mature miRNAs, 2,216 human snoRNAs and scaRNAs and 1,658 human pre-miRNAs. These probe sets were background corrected, normalized and summarized using the Robust Multichip Average (RMA) procedure [59]. The resulting log (base 2) transformed signal intensities were used for ascertaining differentially expressed miRNAs. The significance of the difference in expression between the different treatment groups was ascertained by fitting the data to the one-way ANOVA model, Y_ij_ = μ + Treatment_i_ + Ɛ_ij_, Where Y_ij_ represents the j^th^ observation on the i^th^ treatment group, μ is the common effect for the whole experiment and ε_ij_ represents the random error present in the j^th^ observation on the i^th^ category. The Fisher's Least Significant Difference (FSD) test was used to compare the group means of NVP-BEZ-235, NVP-LDE-225 and the combined NVP-BEZ-235 and NVP-LDE-225, to the Control group. All samples were analyzed in biological duplicates.

### Functional analysis

Biological functional and network analysis of the miRNAs was carried out using the Ingenuity Pathways Analysis software (IPA, Ingenuity Systems, version 7.6 (http://www.ingenuity.com)).

### Generation of Kras^G12D^; *Trp53^LSL-R172H/+^* PDAC mice

Pdx1-Cre mice (generated by Dr. Lowy, University of Cincinnati) were purchased from the Jackson laboratory (Bar Harbor, Maine). LSL-Trp53R172H and LSL-KrasG12D/+ mice were obtained from MMHCC, NCI/NIH. All of these genetically engineered mice were bred and genotyped for the presence of Kras, p53, and Cre [54, 60, 61]. Six-week-old breeding pairs of genetically engineered mice, including transgenic Pdx1-Cre, LSLTrp53 R172H, and LSL-KrasG12D mice were used for breeding. To produce compound transgenic Pan^kras/p53^ mice, the double transgenic LSLKras^G12D/+^-LSL-Trp53^R172/+^ mice were first generated, and then further mated with heterozygous Pdx1-Cre transgenic mice [61]. Pancreatic CSCs were isolated from the Pan^kras/p53^ mice as we described elsewhere [54].

### Antitumor activity of NVP-LDE-225 and/or NVP-BEZ-235 on pancreatic CSC's tumor xenograft in NOD/SCID/IL2Rγ null mice

NOD/SCID/IL2Rϒ null mice were used in this study because these mice are better predictor for the biological response to therapy. Human pancreatic CSCs (1 × 10^6^ cells mixed with Matrigel, Becton Dickinson, Bedford, MA, in 75 μl total volume, 50:50 ratio) were injected subcutaneously into the flanks of 4–6 weeks old NOD/SCID IL2Rγ null mice. After tumor formation, mice were treated with NVP-LDE-225 (40 mg/kg body weight), and/or NVP-BEZ-235 (20 mg/kg body weight), by intraperitoneal injections, 5 times per week for 4 weeks. At the end of the experiment, mice were euthanized, tumors were isolated, measurements were taken and tissues were processed for biochemical analysis.

### Western blot analysis

Pancreatic CSCs treated with NVP-LDE-225 and/or NVP-BEZ-235, and homogenized tumor samples were lysed with RIPA lysis buffer supplemented with protease inhibitor cocktail. Crude protein was resolved on SDS-PAGE gel and transferred onto nitrocellulose membrane (Amersham Biosciences, Piscataway, NJ, USA). After protein transfer, the membrane was blocked with 5% non-fat dry milk, 0.2% Tween 20 in 1x TBS (TBST) for 1 h at room temperature. The membrane was incubated with the primary antibody (1:1000) at 4°C overnight with gentle shaking and washed 3 times with TBST for 15 minutes each. The membrane was incubated with secondary antibody for 1 h at room temperature and then washed 3 times for 15 minutes. The blot was developed with by the addition of ECL substrate (Thermo Fisher Scientific, Rockford, IL).

### Statistical analysis

The mean and SD were calculated for each experimental group. Differences between groups were analyzed by ANOVA Kruskal-Wallis test, with Dunn's multiple group comparison test. *P* values less than 0.05 were considered statistically significant.

### Supplemental information

Supplemental Information includes Supplemental Experimental Procedures.

## SUPPLEMENTARY DATA


