# Supplementary material for: PI3K/AKT/mTOR and sonic hedgehog pathways cooperate together to inhibit human pancreatic cancer stem cell characteristics and tumor growth
Source: Oncotarget. 2015 Oct 5;6(31):32039–60. doi: 10.18632/oncotarget.5055 (PMC4741658; doi:10.18632/oncotarget.5055)
Supplement: Supplementary file 1 [file oncotarget-06-32039-s001.pdf]

SUPPLEMENTARY DATA

EXPERIMENTAL PROCEDURES

Cell viability and apoptosis assays

Cell viability was determined by the XTT assay. Briefly, pancreatic CSCs (5 × 10^4) were incubated with 1 µM of NVP-BEZ-235, 5 µM NVP-LDE-225, and combination of NVP-LDE-225(5 µM) and NVP-BEZ-235(1 µM) in 250 µl of culture medium in 96-well plate for 48 h. 50 µl of freshly prepared XTT-PMS labeling mixture was added to the cell culture. The absorbance was measured at 450 nm with correction at 650 nm. The cell viability was expressed as OD (OD450–OD650).

The apoptosis of pancreatic CSCs was determined by FACS analysis of propidium iodide (PI)-stained cells. Briefly, pancreatic CSCs were grown in 6 well plate and NVP-LDE-225, NVP-BEZ-235 and their combination were added in the media. After 48 h, cells were trypsinized, washed with PBS, resuspended in 200 µl PBS with 10 µl RNAase (10 mg/ml) and incubated at 37*00BA*C for 30 min. After incubation, 50 µl PI solution was added and cells were analyzed for apoptosis using a flow cytometry (Accuri C6 flow cytometer, BD Biosciences, San Jose, CA).

Motility assay

The horizontal motility pancreatic CSCs was examined by using scratch migration assay. Pancreatic CSCs were grown in confluent monolayer and then a scratch is made through the monolayer with a standard 200 µl plastic pipette tip, which gives rise to an *in vitro* wound. After washing twice with PBS, media was replaced in media with or without NVP-LDE-225, NVP-BEZ-235 or their combination. Pancreatic CSCs migrate into the scratch area as single cells from the confluent sides. The width of the scratch gap is viewed under the microscope in four separate areas each day until the gap is completely filled in the untreated control wells. Three replicate wells from a six-well plate were used for each experimental condition.

Transwell migration assay

In order to examine transwell migration assay, 1 × 10^5 pancreatic CSCs were plated in the top chamber onto the non-coated membrane (24-well insert; pore size, 8 mm; Corning Inc.) and allowed to migrate in the lower chamber towards serum-containing medium. After 24 h of incubation, cells were fixed with methanol and stained with Diff-Quick Fixative Solutions (Dade Behring, Newark, DE, USA).

Tumor spheroid assay and Immunocytochemistry

For spheroid forming assay, CSCs were grown in six-well ultralow attachment plates (Corning Inc.) at a density of 1000 cells/ml in DMEM supplemented with 1% N_2 (Life Technologies), 2% B27 (Life Technologies), 20 ng/ml human platelet growth factor (Sigma-Aldrich, St Louis, MO, USA), 100 ng/ml epidermal growth factor (Life Technologies) and 1% antibiotic-antimycotic (Life Technologies) at 37ºC in a humidified atmosphere of 95% air and 5% CO_2. NVP-LDE-225 (5 µM), NVP-BEZ-235 (1 µM) or their combination was added to cells during the formation of spheroids. Similarly, for secondary and tertiary spheroids, CSCs from spheroids were treated with drugs and grown for 7 days.

For immunocytochemistry, primary spheroids treated with drugs were fixed with 4% paraformaldehyde for 15 min, permeabilized with 0.1% Triton X-100 in PBS, washed and blocked in 10% normal goat serum. After washing with PBS, cells were stained with Nanog, Sox2, Oct4, and c-Myc primary antibodies (1:100) for 16 h at 4ºC and washed with PBS. Afterwards, cells were incubated with fluorescently labeled secondary antibody (1:200) along with DAPI (1 mg/ml) for 1 h at room temperature. Finally, coverslips were washed and mounted using Vectashield (Vector Laboratories, Burlington, CA, USA). Isotype-specific negative controls were included with each staining. Stained spheroids of pancreatic CSCs were mounted and visualized under a fluorescent microscope. Imunohistochemistry of pancreatic tumor tissues was performed.

RNA isolation and mRNA expression analysis by qRT-PCR

Total RNAs were isolated using the trizol reagent (invitrogen, CA, USA). Complementary DNAs were synthesized by oligo(dT)-priming methods. Real-time PCR was performed using the SYBR Green Supermix (Qiagen) according to the manufacturer’s instructions. Primers specific for each of the signaling molecules were designed using NCBI/Primer-BLAST and used to generate the PCR products. Expression levels of glyceraldehyde-3-phosphate dehydrogenase (GAPDH) were used for normalization and quantification of gene expression levels. For the quantification of gene amplification, real-time PCR was performed using an ABI 7300 Sequence Detection System in the presence of SYBR-Green. The following gene specific primers were used:

Patched1 (Fw, TGACCTAGTCAGGCTGGAAG; Rv, GAAGGAGATTATCCCCCTGA)

Patched-2 (Fw, AGGAGCTGCATTACACCAAG; Rv, CCCAGGACTTCCCATAGAGT)

Gli1 (Fw, CTGGATCGGATAGGTGGTCT; Rv, CAGAGGTTGGGAGGTAAGGA)

Gli2 (Fw, GCCCTTCCTGAAAAGAAGAC; Rv, CATTGGAGAAACAGGATTGG)

Myc (Fw, CGACGAGACCTTCATCAAAA; Rv, TGCTGTCGTTGAGAGGGTAG)

Nanog (Fw, ACCTACCTACCCCAGCCTTT; Rv, CATGCAGGACTGCAGAGATT)

Sox-2 (Fw, AACCCCAAGATGCACAACTC; Rv, GCTTAGCCTCGTCGATGAAC)

Oct-4 (Fw, GGACCAGTGTCCTTTCCTCT; Rv, CCAGGTTTTCTTTCCCTAGC)

Snail (Fw, ACCCCACATCCTTCTCACTG; Rv, TACAAAAACCCACGCAGACA)

Slug (Fw, ACACACACACACCCACAGAG; Rv, AAATGATTTGGCAGCAATGT)

Zeb1 (Fw, GCACAACCAAGTGCAGAAGA; Rv, CATTTGCAGATTGAGGCTGA)

LIN28 (Fw, GACCCAAAGGGAAGACACTA; Rv, TCTTCCCTGAGAACTCGCGG)

HK-GAPD (Fw, GAGTCAACGGATTTGGTCGT; Rv, TTGATTTTGGAGGGATCTCG)

MicroRNA array analysis

The Genomics Core utilized the Affymetrix GeneChip system for processing miRNA expression arrays. 1ug of total RNA was labeled using the FlashTag Biotin HSR RNA Labeling Kit (Affymetrix #901910). The FlashTag labeling system employed the 3DNA dendrimer signal amplification technology. 3DNA dendrimer is a branched structure of single and double stranded DNA conjugates which incorporates numerous biotin labels for ultrasensitive expression detection. Using the GeneChip 645 Hybridization Oven, labeled target RNA was hybridized overnight to interrogating oligo probes contained in the GeneChip miRNA 3.0 expression cartridge array (Affymetrix #902017). Hybridized Genechips underwent low and high stringency washing and R-Phycoerythrin-Streptavidin staining procedures using the GeneChip fluidics station 450. After washing, GeneChips underwent a single scan using a GeneChip scanner 3000 7G with autoloader. Fluidics and scan functions were controlled by Affymetrix GeneChip Command Console software (AGCC). Raw expression data was loaded onto the Microarray Data Management System (MDMS) for further data analysis.

The expression levels of expressed miRNAs were measured using Affymetrix GeneChip miRNA 3.0 arrays. Only probesets of human small-RNAs in the array were used for the analysis. They constituted 1,733 human mature miRNAs, 2,216 human snoRNAs and scaRNAs and 1,658 human pre-miRNAs. These probesets were background corrected, normalized and summarized using the Robust Multichip Average (RMA) procedure {Irizarry, 2003 #4870}. The resulting log (base 2) transformed signal intensities were used for ascertaining differentially expressed miRNAs. The significance of the difference in expression between the different treatment groups was ascertained by fitting the data to the one-way ANOVA model, $Y_{ij} = \mu + Treatment_i + \mathcal{E}_j$, Where Y_{ij} represents the jth observation on the ith treatment group, μ is the common effect for the whole experiment and ε_{ij} represents the random error present in the jth observation on the ith category. The Fisher's Least Significant Difference (FSD) test was used to compare the group means of NVP-BEZ-235, NVP-LDE-225 and the combined NVP-BEZ-235 and NVP-LDE-225, to the Control group. All samples were analyzed in biological duplicates.

Functional analysis

Biological functional and network analysis of the miRNAs was carried out using the Ingenuity Pathways Analysis software (IPA, Ingenuity Systems, version 7.6 (http://www.ingenuity.com)).

Generation of Kras^{G12D}; *Trp53*$^{LSL-R172H/+}$ PDAC mice

Pdx1-Cre mice (generated by Dr. Lowy, University of Cincinnati) were purchased from the Jackson laboratory (Bar Harbor, Maine). LSL-Trp53R172H and LSL-KrasG12D/+ mice were obtained from MMHCC, NCI/NIH. All of these genetically engineered mice were bred and genotyped for the presence of Kras, p53, and Cre. Six-week-old breeding pairs of genetically engineered mice, including transgenic Pdx1-Cre, LSLTrp53 R172H, and LSL-KrasG12D mice were used for breeding. To produce compound transgenic Pan$^{kras/p53}$ mice, the double transgenic LSLKras$^{G12D/+}$-LSL-Trp53$^{R172/+}$ mice were first generated, and then further mated with heterozygous Pdx1-Cre transgenic mice. Pancreatic CSCs were isolated from the Pan$^{kras/p53}$ mice.

Antitumor activity of NVP-LDE-225 and/or NVP-BEZ-235 on pancreatic CSC's tumor xenograft in NOD/SCID/IL2Rγ null mice

NOD/SCID/IL2Rϒ null mice were used in this study because these mice are better predictor for the biological response to therapy. Human pancreatic CSCs (1×10^6 cells mixed with Matrigel, Becton Dickinson, Bedford, MA, in 75 µl total volume, 50:50 ratio) were

injected subcutaneously into the flanks of 4–6 weeks old NOD/SCID IL2Rγ null mice. After tumor formation, mice (7 mice per group) were treated with NVP-LDE-225 (40 mg/kg body weight), and/or NVP-BEZ-235 (20 mg/kg body weight), by intraperitoneal injections, 5 times per week for 4 weeks. At the end of the experiment, mice were euthanized, tumors were isolated, measurements were taken and tissues were processed for biochemical analysis.

Western blot analysis

Pancreatic CSCs treated with NVP-LDE-225 and/or NVP-BEZ-235, and homogenized tumor samples were lysed with RIPA lysis buffer supplemented with protease inhibitor cocktail. Crude protein was resolved on SDS-PAGE gel and transferred onto nitrocellulose membrane (Amersham Biosciences, Piscataway, NJ, USA). After protein transfer, the membrane was blocked with 5% non-fat dry milk, 0.2% Tween 20 in 1x TBS (TBST) for 1 h at room temperature. The membrane was incubated with the primary antibody (1:1000) at 4°C overnight with gentle shaking and washed 3 times with TBST for 15 minutes each. The membrane was incubated with secondary antibody for 1 h at room temperature and then washed 3 times for 15 minutes. The blot was developed with by the addition of ECL substrate (Thermo Fisher Scientific, Rockford, IL).
